# Role of myeloid cells in ischemic retinopathies: recent advances and unanswered questions

**DOI:** 10.1186/s12974-024-03058-y

**Published:** 2024-03-07

**Authors:** Rami A. Shahror, Carol A. Morris, Aya A. Mohammed, Melissa Wild, Bushra Zaman, Christian D. Mitchell, Paul H. Phillips, Nancy J. Rusch, Esraa Shosha, Abdelrahman Y. Fouda

**Affiliations:** 1 Department of Pharmacology and Toxicology, College of Medicine, University of Arkansas for Medical Sciences (UAMS), 4301 West Markham Street, Slot 611, BIOMED-1, B306, Little Rock, AR 72205 USA; 2 Department of Ophthalmology, Harvey & Bernice Jones Eye Institute, University of Arkansas for Medical Sciences, Little Rock, AR USA; 3 Clinical Pharmacy Department, Cairo University, Cairo, Egypt

**Keywords:** Retinopathy, Retinal ischemia, Diabetic retinopathy, Retinopathy of prematurity, Oxygen-induced retinopathy, Myeloid cells, Microglia, Macrophages

## Abstract

Myeloid cells including microglia and macrophages play crucial roles in retinal homeostasis by clearing cellular debris and regulating inflammation. These cells are activated in several blinding ischemic retinal diseases including diabetic retinopathy, where they may exert both beneficial and detrimental effects on neurovascular function and angiogenesis. Myeloid cells impact the progression of retinal pathologies and recent studies suggest that targeting myeloid cells is a promising therapeutic strategy to mitigate diabetic retinopathy and other ischemic retinal diseases. This review summarizes the recent advances in our understanding of the role of microglia and macrophages in retinal diseases and focuses on the effects of myeloid cells on neurovascular injury and angiogenesis in ischemic retinopathies. We highlight gaps in knowledge and advocate for a more detailed understanding of the role of myeloid cells in retinal ischemic injury to fully unlock the potential of targeting myeloid cells as a therapeutic strategy for retinal ischemia.

## Introduction

The retina comprises the light-sensing layer of the eye. It relies on a delicate interplay between neuronal function, vascular supply, and immune surveillance for normal function. In recent years, myeloid cells that include microglia and macrophages have emerged as crucial players in maintaining retinal homeostasis. These cells can exert a trophic influence on neuronal development in the retina and serve as sentinels of the microenvironment to clear microorganisms and cell debris. Conversely, these same cells can participate in diverse retinal pathologies as evidenced by their dynamic ability to switch between different functional phenotypes. For example, microglia can exert detrimental effects on the compromised retina by releasing a wide range of cytokines that can exacerbate the underlying condition.

The purpose of this review is to summarize and highlight these emerging and sometimes confounding roles of the myeloid cells in the retina with a particular focus on ischemic retinopathies. We initially will provide an anatomical and physiological foundation for understanding the retina, including an overview of retina structure, blood supply, the blood-retina barrier, and the retina neurovascular unit. Subsequently, we will address the origin and types of retina myeloid cells, differentiating between resident microglia and infiltrating macrophages. Further discussion will focus on the cutting-edge tools and methods available for defining the dynamic roles of the myeloid cells in the retina. The ensuing discussion will delve into the diverse phenotypes and functional specialization of myeloid cells in ischemic retinopathies and neurodegeneration, diabetic retinopathy, and anomalous retinal angiogenesis. We will conclude by highlighting knowledge gaps in our understanding of the roles of myeloid cells in the retina and raise unanswered questions in the field that are promising avenues for future research.

In the preparation of this review, biomedical literature was searched comprehensively using the electronic National Institutes of Health’s PubMed^®^ database and National Library of Medicine’s MEDLINE database. The search strategy included a cross-referencing of the following terms: ischemic retinopathy, retinal ischemia–reperfusion injury, or retinopathy of prematurity, and myeloid cells, microglia, or macrophages. Regrettably, we had to exclude published papers with text language other than English. Our review is intended to provide a valuable resource for both experts and newcomers to the retinal field and everyone else seeking to understand the complex role of myeloid cells in retinal health and disease.

## Structure and function of the retinal neurovascular unit

### Retinal layers

**Fig. 1:**
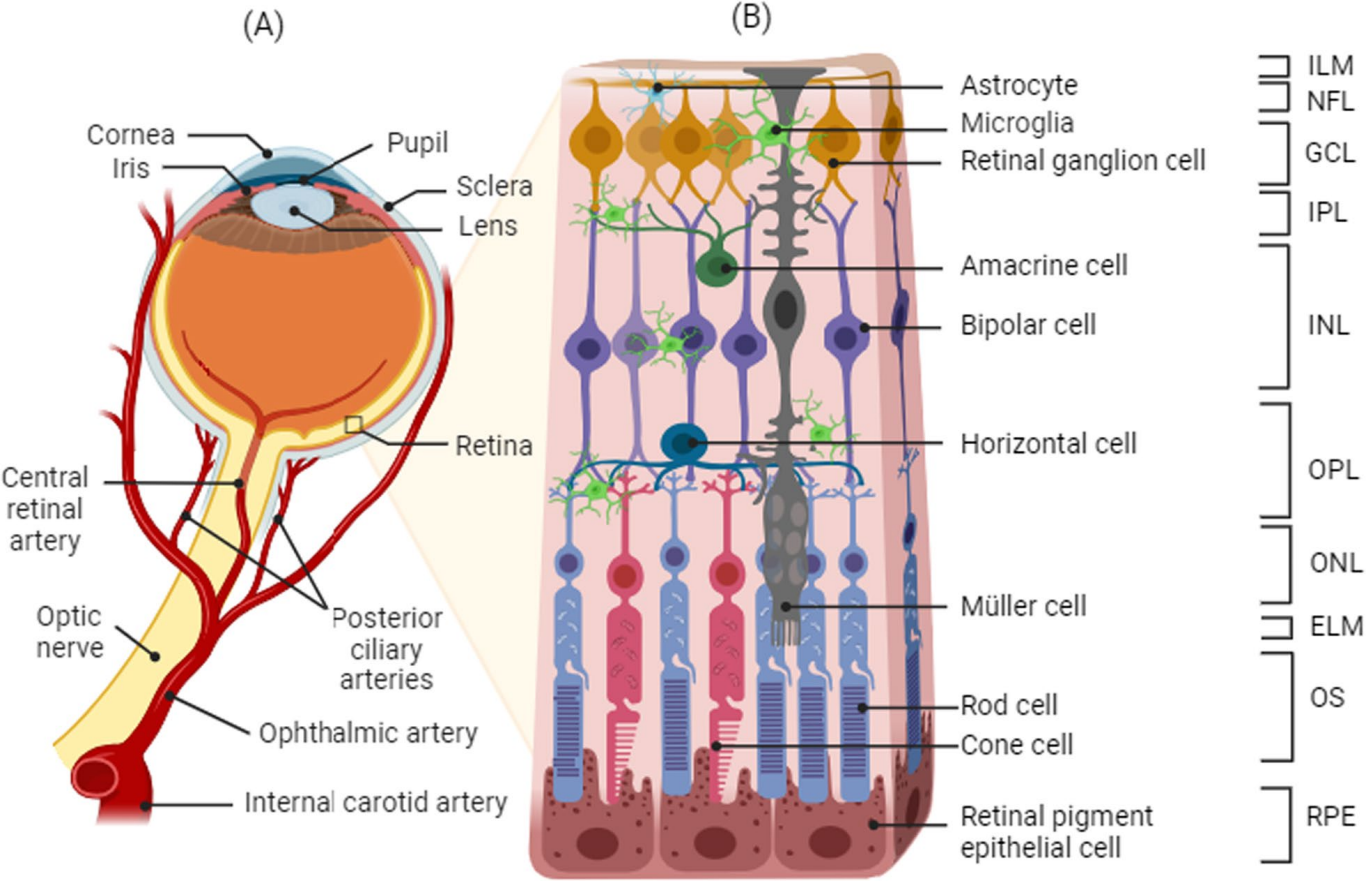
**A** Blood supply and **B** structure of the retina. ILM: internal limiting membrane, NFL: nerve fiber layer, GCL: ganglion cell layer, IPL: inner plexiform layer, INL: inner nuclear layer, OPL: outer plexiform layer, ONL: outer nuclear layer, ELM: external limiting membrane, OS: photoreceptor outer segment, RPE: retinal pigment epithelium

The retina is the innermost structure of the eye that converts light into electrical and chemical signals transmitted by the optic nerve to the brain’s occipital lobe to form high-resolution images [1, 2]. The structure of the retina includes ten interconnected layers of cells, each of which is critically important for retinal function (Fig. 1). The innermost layer (farthest from the optic nerve) is the *Inner Limiting Membrane* (ILM) that separates the retina and the vitreous cavity. It is considered a basement membrane and serves as the basal lamina for the ganglion cell axons, astrocytes, and Müller glial cells. The second innermost layer of the retina is the *Nerve Fiber Layer* (NFL). The NFL is composed of retinal vessels, glial cells, and the axons of retinal ganglion cells (RGCs) that collectively form the optic nerve [3]. The RGC nuclei are situated in the *Ganglion Cell Layer* (GCL), the third layer, and primarily interspersed with vessel cells, glial cells, and some displaced amacrine cells. Fourthly, the *Inner Plexiform Layer* (IPL) is the site of interaction between bipolar, amacrine, and ganglion cells. The nuclei from bipolar, horizontal, amacrine, and Müller cells are located in the fifth layer, the *Inner Nuclear Layer* (INL). The sixth layer is the *Outer Plexiform Layer* (OPL) where photoreceptor cells connect with bipolar cells, and where horizontal cells interact closely with both photoreceptors and bipolar cells. Nuclei from photoreceptor cells are found in the seventh layer, the *Outer Nuclear Layer* (ONL). Eighthly, the *External Limiting Membrane* (ELM) is created by junctional complexes between adjacent Müller cells, and between Müller and photoreceptor cells. Ninthly, the *Outer Segment* (OS) contains tightly stacked outer segments of the photoreceptors cones and rods forming a palisading layer. Finally, the tenth layer is the *Retinal Pigmented Epithelium* layer (RPE) which is a monolayer of retinal pigmented epithelial cells joined by tight junctions [4].

Incoming light passes through the retina layers until it reaches and activates the photoreceptors, the light sensitive neurons. The photoreceptors (rods and cones) send information to bipolar cells that form synapses with amacrine cells and RGCs to process electrical signals in the inner retina. Subsequently, the RGCs propagate visual stimuli to the brain by transmitting the signal from the retina through their axons in the optic nerve.

### Retinal blood supply and the blood-retinal barrier

The retina is highly metabolically active, and it requires an extensive blood supply and capillary network to furnish adequate oxygen and nutrients. A dual blood supply coupled to extensive anastomoses ensure that the retinal demands for high oxygen consumption are met under normal conditions (Fig. 1). The central retinal artery originates from the ophthalmic artery, a branch of the internal carotid artery, and travels within the optic nerve sheath to supply blood to the inner two-thirds of the retina. It forms three distinct layers of anastomosing blood vessels that include the superficial vascular plexus located in the NFL and the intermediate and deep vascular plexuses found on each side of the INL layer [5, 6]. The posterior ciliary arteries supply the choroid, RPE, outer retina, and the optic nerve head. Ultimately, blood is returned from the retinal capillaries to larger veins outside the eye by the carotid vein, which also travels within the optic nerve tract.

**Fig. 2:**
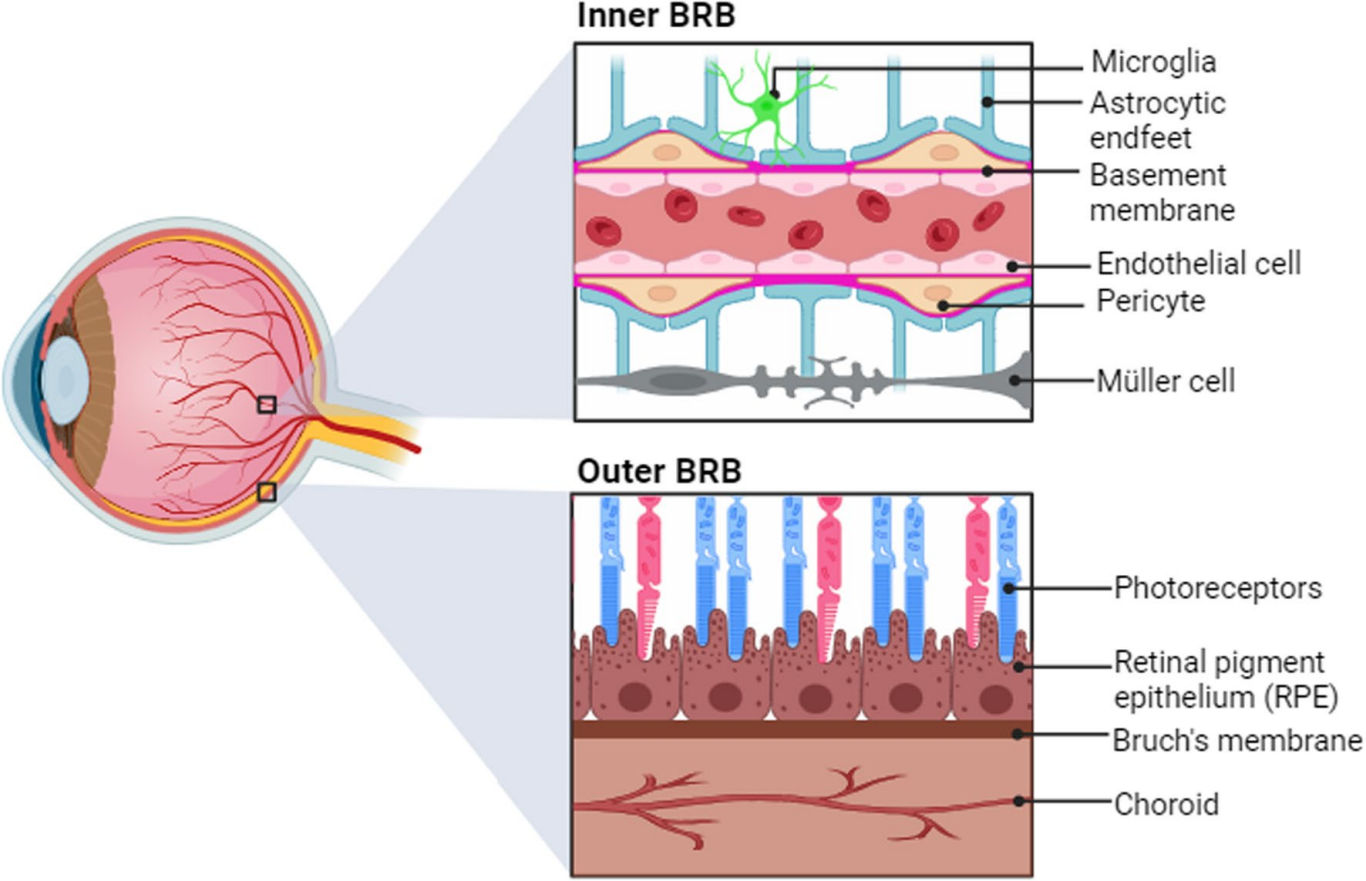
Anatomical illustration depicting the locational and structural differences between the inner BRB (top) and the outer BRB (bottom)

The retina arises from the forebrain during fetal development [3, 7], and with its populations of neurons, glia, and vascular cells, it shares many features with the rest of the central nervous system (CNS). For example, the retina and brain are both critical structures that must carefully titrate the arrival of oxygen and nutrients to optimize the function of their metabolically active cells. Accordingly, a blood-retinal barrier (BRB) tightly regulates the movement of substances from blood to retinal cells analogous to the function of the blood brain barrier (BBB). However, unlike the single layer of cerebral endothelial cells that compose the BBB, the BRB has two layers known as the inner and outer BRB (Fig. 2) [8]. The inner BRB resembles the BBB in that endothelial cell tight junctions prevent the movement of large molecules from the blood into the inner retina. In addition to endothelial cells, the inner BRB also is home to other neurovascular cells including pericytes, astrocytes, Müller cells, and microglia. In contrast, the outer BRB is formed by the RPE and Bruch's membrane. The latter is an extracellular matrix located between the RPE and the choroidal capillaries of the eye that acts as an additional molecular sieve for selective bi-directional exchange of oxygen and nutrients between the retinal and choroid circulation to maintain retinal homeostasis [9].

### The retinal neurovascular unit

The concept of a *Neurovascular Unit* (NVU) was introduced to describe the functional interaction between neurons and their blood supply in the CNS. This term was adapted to describe a similar phenomenon of “neurovascular coupling” in retina by which neurons signal through glial cells to regulate vascular tone, and thereby titrate blood flow to match the intensity of neuronal activity [10]. Based on the retinal anatomy discussed earlier, we can conclude that three types of cells fundamentally comprise the NVU in retina: (1) neurons of various subtypes such as ganglion cells, bipolar cells, horizontal cells, amacrine cells, and photoreceptors; (2) vascular cells including endothelial cells, smooth muscle cells, and pericytes; and (3) supporting glial cells consisting of Müller cells, astrocytes, microglia, and other immune cells [1].

**Fig. 3:**
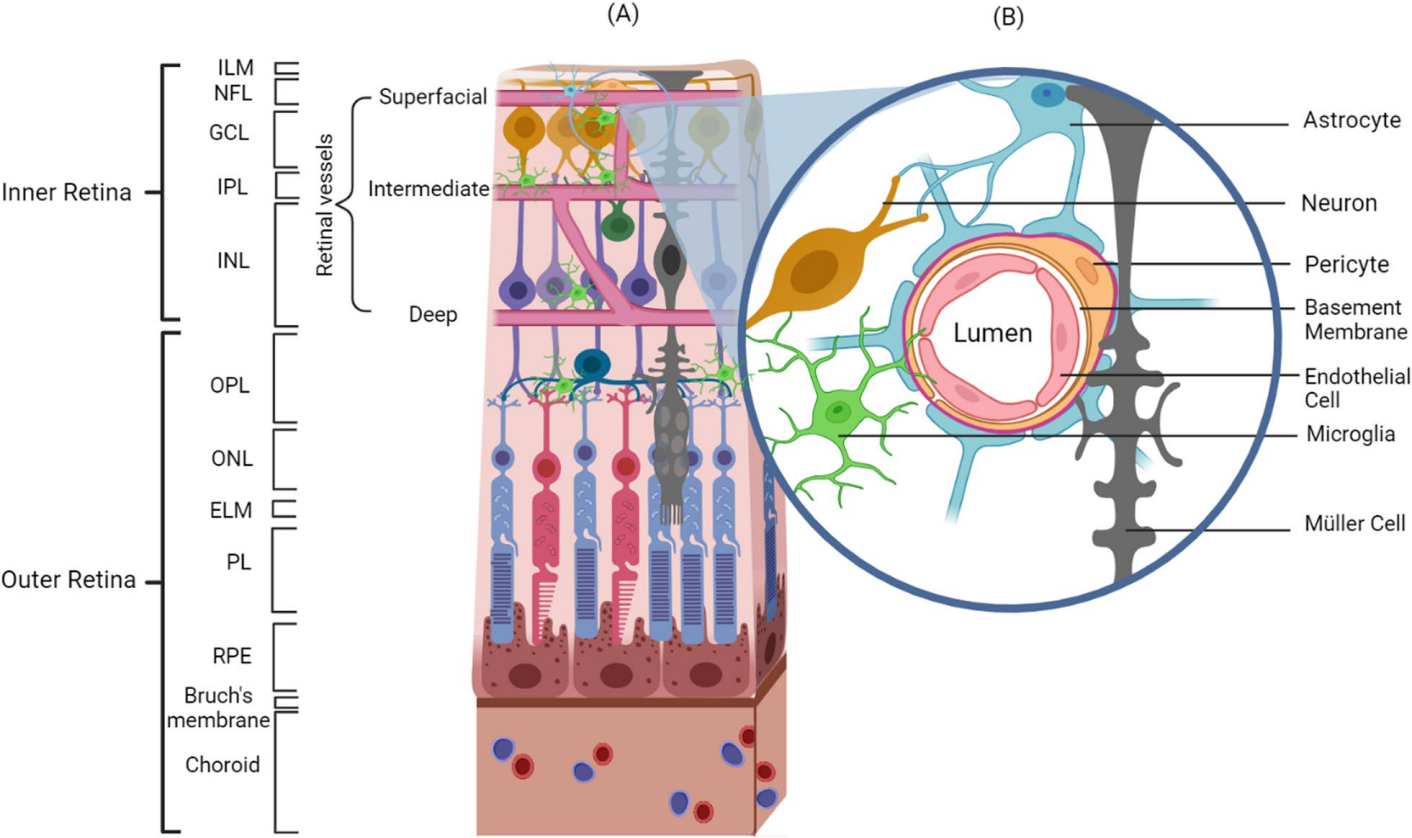
**A** Depiction of the retinal neurovascular unit (NVU) showing the anatomical arrangement of retinal cell layers and the retinal blood vessels composing the superficial, intermediate, and deep vascular plexuses. **B** The composition of the retinal NVU

Although these three categories of cells (e.g., neurons, vascular, glial) are the building blocks of the NVU, the subtypes of participating cells differ based on the layer of the retina. Fundamentally, however, all cells of the retinal NVU follow a basic structural arrangement whereby the neurons communicate with the astrocytes, and the astrocytes frame the vasculature. An important feature of the vascular wall is the endothelial cell layer, which lines the vessel lumen and features tight junctions that limit endothelial permeability; the endothelium is encircled by the basement membrane. Pericytes also wrap around the retinal capillaries at discrete points on the basement membrane and regulate permeability, blood flow, and other microvascular functions. The microenvironment of the NVU also contains other types of glial cells including microglia and Müller cells, which constitute the majority of the support glial cells in the retina (Fig. 3). In order for the NVU to function properly, it needs a richly balanced environment of ions, neurotransmitters, and energy in the form of adenosine triphosphate (ATP), which is mediated by the extracellular matrix (ECM). The ECM is composed of different forms of glycoproteins such as collagen, fibronectin, and laminin that compose the basement membrane [11]. Ultimately, the NVU orchestrates the maintenance of the BRB integrity and retinal homeostasis.

### Retina as an immune-privileged organ

Another similarity between the retina and CNS is that both organs are considered immune-privileged sites, a term that refers to their ability to conduct active surveillance to limit the entry of systemic immune cells. The retina implements strategies including avoidance, tolerance, and resistance to combat pathogens. The blood-retina barrier provides a physical barrier that reduces the risk of exposure of the retina to infiltrating pathogens. Other lines of defense are the immune privileged sites in the intraocular compartment, such as the vitreous cavity, subretinal space, and anterior chamber. These sites promote tolerance instead of rejection of infiltrating pathogens, and instead of actively reducing pathogen numbers, they exert anti-inflammatory and immune-modulatory mechanisms to buffer pathogen-induced injury. The retina can also activate resistance mechanisms which involve activation of myeloid cells and the complement system. Collectively, these lines of defense offer layered protection to the retina so that even when the BRB is disrupted, tolerance and resistance can provide protection and reduce injury [12].

As inferred above, the retina and CNS have their own tissue-resident myeloid cells that include microglia and resident macrophages that are distinct from the immune cells of peripheral blood. In response to injury or neurovascular degeneration, the resident myeloid cells become activated and demonstrate either pro-inflammatory or anti-inflammatory phenotypes, which are triggered by specific cues derived from the local microenvironment of the retina [13]. The role of myeloid cells in the outer retina has been detailed in excellent recent reviews [14–16]. The remaining sections of this review provide an updated perspective on the roles of myeloid cells in angiogenesis and neurodegeneration in the inner retina and experimental approaches to delineate their functions in health and disease.

## Origin and types of retinal myeloid cells

Mononuclear phagocytic cells (MPs) in the retina consist of parenchymal myeloid cells located within the tissue that are associated with primary retinal cells, and myeloid cells associated with nonparenchymal tissues including the vascular and blood components of the retinal structure. In general, the MPs in the retinal can be classified as (1) microglia that reside in the parenchyma; (2) nonparenchymal border-associated resident macrophages including perivascular macrophages [17, 18]; and (3) blood-derived monocytes that infiltrate the retina in response to injury and differentiate into macrophages [19]. Both microglia and macrophages participate in the maintenance of tissue homeostasis in the retina but they also may contribute to disease as described in later sections [20, 21].

### Microglia

Microglia, the resident immune cells of the CNS, are predominantly located in the IPL and OPL layers of the retina. They also can be found in the GCL and INL (but not the ONL) of the retina during normal physiological conditions [22]. These microglia are derived from the embryonic yolk sac and play a critical role in neuronal pruning and the formation of angiogenesis networks during development [18]. In the healthy retina, microglia are active surveyors of their microenvironment and continuously modify the length of their processes to explore their surroundings and dynamically promote homeostasis in the retinal tissues [23–25]. Additionally, microglia are involved in neuronal excitability, synaptic organization, and trophic neuronal support during development [26]. The retinal microglia additionally survey the local milieu to clear microorganisms and damaged cells [27].

When resident microglia detect homeostatic disruptions of the retina, such as the presence of pathogens divulged by pathogen-associated molecular patterns (PAMPS), or the disruption of the BRB revealed by danger-associated molecular patterns (DAMPs), they retract their processes to increase their mobility and undergo phenotypic transformation [25]. After activation in response to sterile or infectious insults, the microglia begin to release cytokines that amplify the inflammatory response. Microglia also engage in phagocytosis during physiological and pathophysiological conditions as described later in this review.

### Perivascular macrophages and capillary-associated microglia

**Fig. 4:**
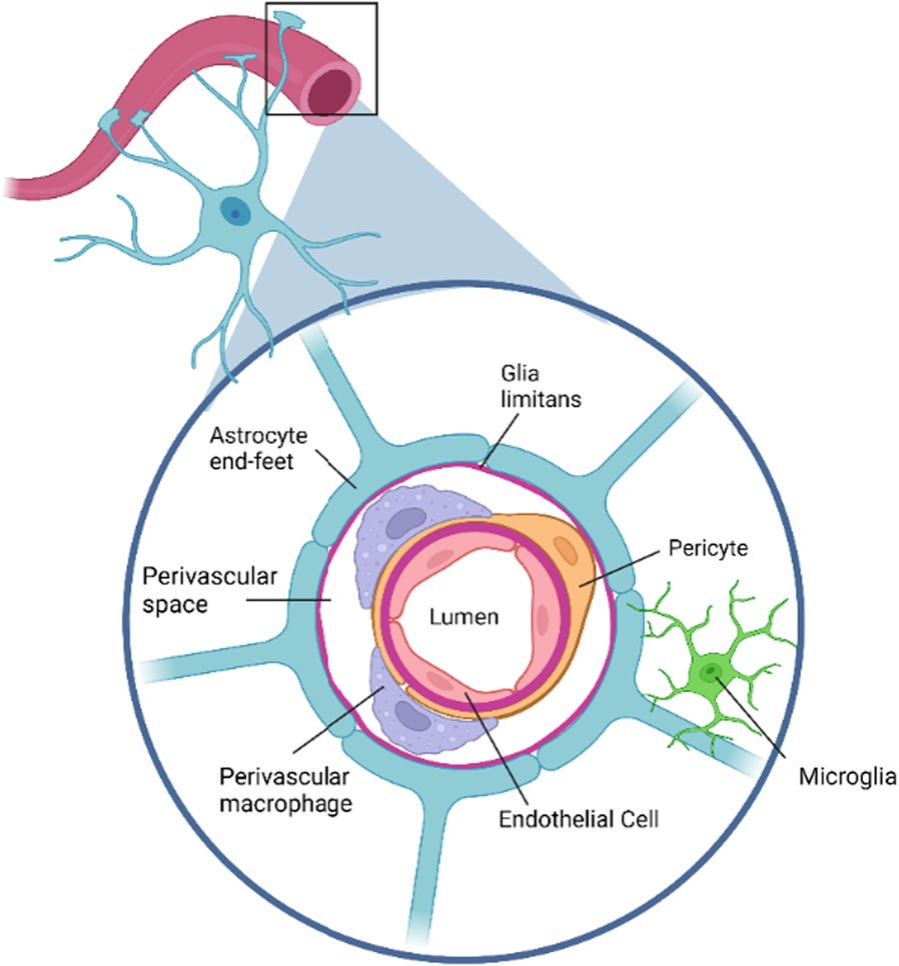
Illustration of the perivascular macrophages (PVM) within the perivascular space

Perivascular macrophages (PVM) are specialized macrophages that surround the blood vessels of the retina and CNS (Fig. 4). They are identified by their vicinity to the vasculature and high expression of the CD206, CD163, and CD45 markers, whereas expression of the microglial marker Iba-1 is low [28]. PVMs are located in the perivascular space formed between the retinal microvessel wall and the glia limitans (glial limiting membrane that is made up of astrocytic foot processes), and they help to maintain the integrity of the blood–brain barrier by phagocytosing cellular debris and foreign particles as well as antigen presentation [29]. Unlike the brain [30], only a limited number of studies have sought to define the role of PVMs in the retina. One report showed that the scavenger function of PVMs may promote the BRB integrity in the retina similar to the known scavenger benefit of PVMs in the CNS [31]. This study demonstrated the migration of perivascular macrophages to the site of injury and BRB breakdown after intravitreal injection of mannitol. However, the authors did not directly test the role of the PVMs in barrier integrity. Another study documented PVM recruitment to the injury site caused by experimental retinal vein occlusion in mice and argued for a protective role of PVM since their depletion increased endothelial cell apoptosis [32].

Emerging studies reveal complex and dynamic interactions between microglia and the retinal vasculature that involve multiple microglia subtypes, localized functions, and cell-to-cell interactions between different vascular components. For example, a recent study using electron microscopy identified a population of juxtavascular microglia (JVM) capable of phagocytosing dying pericytes in diabetic human and dog retinas [33]. The JVM constituted an integral part of the glia limitans and were regarded as distinct from the PVM that reside in the vascular space (vascular side of the glia limitans) [33]. Another report identified capillary-associated microglia (CAMs) that assisted in retinal tissue homeostasis by clearing debris during vascular pruning to enable critical remodeling during development of the retinal circulation [34]. Interestingly, CAMs also exert a physiological effect on retinal capillaries by directly contacting and communicating with endothelial cells. For example, the chemokine fractalkine stimulates its receptor, CX3CR1, on microglia to initiate vasoconstriction at direct contact sites of microglial processes [35]. Clearly, a more complete characterization of the role of perivascular macrophages and vessel-associated microglia is essential to clarify their diverse roles in retinal homeostasis and BRB integrity.

### Blood-derived monocytes/macrophages

Infiltrating myeloid cells including circulating or blood-borne monocytes and macrophages are recruited to the retina in response to injury to participate in the immune response [36–38]. These circulating myeloid cells must traverse the BRB to respond effectively to the retinal injury. Still, this event is curtailed by the retina as an “immune-privileged” organ in an effort intended to protect visual function [39]. Fundamentally, the tight junctions between the retinal capillary endothelial cells prevent myeloid cell penetration across the BRB [40]. However, this barrier can be penetrated with the help of adhesion molecules including vascular cell adhesion molecule-1 (VCAM-1), intercellular adhesion molecule-1 (ICAM-1), and platelet-endothelial cell adhesion molecule-1 (PECAM-1). These adhesion molecules allow the circulating myeloid cells to adhere to the endothelium and assist in their paracellular migration [41, 42]. Less commonly, circulating myeloid cells may penetrate the BRB by *trans*cellular migration, a process in which a myeloid cell initially interacts with an adhesion molecule such as VCAM-1 or ICAM-1 on endothelial cells prior to being endocytosed into the endothelial cells and subsequently transported by transcytosis across the BRB [43, 44].

## Tools to study myeloid cells in models of retinal disease

**Fig. 5:**
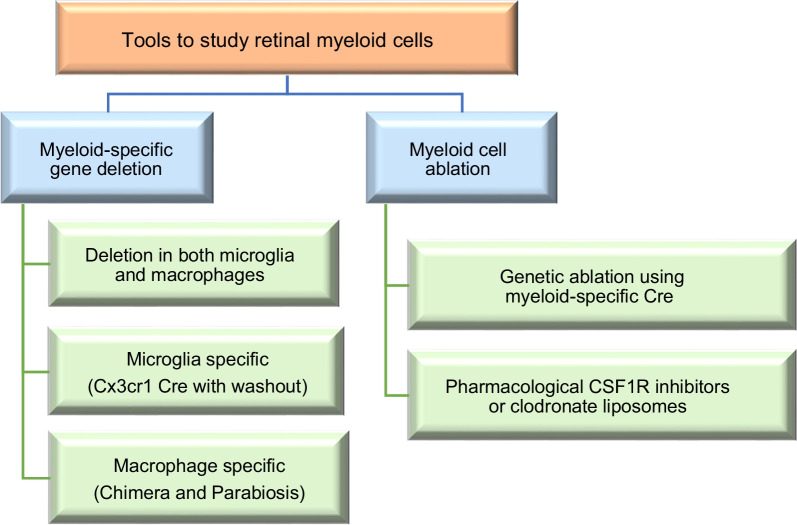
Diagram showing the different tools used to study retinal myeloid cells

Tools to study the myeloid cell response in retinal diseases include myeloid-specific deletion of genes of interest using the Cre-lox system and the ablation of myeloid cells either genetically or pharmacologically (Fig. 5). These approaches are discussed below.

### Genetic models of myeloid-specific KO mice

Myeloid-specific Cre drivers are employed to study the role of a specific gene in the myeloid cell response to retinal diseases. LysM Cre has been widely used to delete genes in macrophages and microglia [45, 46]. We recently reported that LysM Cre effectively targets macrophages but is only expressed in 30% of microglia at resting state and shows residual neuronal expression as well [47, 48]. Another useful Cre driver that targets both macrophages and microglia is Cx3cr1 [49–51]. A tamoxifen (TAM)-inducible Cx3cr1 Cre is usually employed to avoid possible neuronal recombination in constitutive Cx3cr1 Cre mice and is now considered the standard choice for targeting myeloid cells in retina research [52].

Unfortunately, these Cre drivers do not differentiate between microglia and infiltrating bone marrow-derived macrophages. Fate-mapping has been utilized to overcome this problem. Using this strategy, microglia-specific gene deletion can be achieved by including a washout period of 8 weeks after TAM administration to the Cx3cr1 mice to allow turnover of shorter-lived circulating monocytes while the gene of interest remains knocked out in microglia that have a longer half-life and are self-renewing [49, 53]. Conversely, macrophage-specific deletion can be achieved using bone marrow chimeras or parabiosis. In order to achieve bone marrow chimeras, recipient mice are lethally irradiated to ablate bone marrow cells before receiving donor mice bone marrow cells by tail vein injection [49]. However, radiation per se can lead to blood-retina barrier breakdown and immune cell recruitment and activation [54]. Using a head shield can protect the BRB from radiation-induced damage but may not achieve full blood chimerism [15]. Parabiosis is achieved by the surgical conjoining of two mice to develop a joined circulatory system. The two mice express distinct leukocyte cell surface markers (CD45.1, CD45.2) that can be differentiated via flow cytometry. Alternatively, one of them is a reporter mouse with fluorescent myeloid cells so the experimenter can determine if the immune cells responding to retinal injury in the non-reporter mouse are derived from the blood circulation [55]. This approach circumvents the toxic radiation effects. However, it only achieves ~ 50% chimerism since the immune cell of the original mouse are not depleted and therefore the contribution of systemic myeloid cells to injury outcomes may be underestimated [56]. Parabiosis is also technically challenging and requires a recovery period after surgery.

In addition to gene-specific deletion, the myeloid-cell targeting methods described above can be coupled with reporter mice expressing green fluorescent protein (GFP) or the red fluorescent protein td tomato to trace the origin of myeloid cells responding to a retinal injury.

### Pharmacological/genetic approaches to induce microglia/macrophage depletion

#### Genetic ablation

A genetic strategy used to induce microglia/macrophage depletion is to employ a diphtheria toxin (DT) -inducible system using the above-mentioned myeloid Cre drivers [57]. After TAM administration, the diphtheria toxin receptor (DTR) is expressed by excision of a STOP cassette in myeloid Cre-expressing cells, which renders them susceptible to diphtheria toxin treatment. The DT-inducible system achieves > 80–90% depletion of the target gene by 2 to 3 days after DT treatment. Depletion is transient and reversed by 7 days after DT discontinuation. Treatment with DT leads to the termination of protein synthesis and apoptotic cell death of myeloid cells expressing DTR. Since DT crosses the blood–brain and retina barriers, systemic treatment promotes cell ablation in the CNS and peripheral circulation. Available DT-inducible mice include CD11c^DTR^, and CX3CR1^CreER^:R26^iDTR^ [58, 59]. As described above, including a washout period of 4 to 6 weeks between TAM treatment and DT administration in the CX3CR1^CreER^:R26^iDTR^ mice can enable specific depletion of microglia while sparing peripheral macrophages. Furthermore, local cell depletion can be achieved using intravitreal DT treatment. However, the duration of depletion is short due to the limited number of times that intravitreal injections can be administered in a short time period to mice.

#### Pharmacological ablation

In addition to the genetic ablation models discussed above, myeloid cells can be ablated pharmacologically, which eliminates the need for breeding and genotyping associated with genetic ablation.

Pharmacological ablation of microglia can be achieved by oral administration of small molecule inhibitors of the receptor of the colony-stimulating factor-1 (CSF1R), PLX-3397, and a related CSF1R inhibitor PLX-5622. CSF1R inhibitors effectively cross the blood-retina barrier leading to nearly complete microglial depletion. Microglial depletion by these drugs that can be formulated into rodent chow diet or given as oral gavage tends to be slow, requiring 3 to 7 days after administration of PLX-5662 and as long as 3 weeks after initiation of PLX3397 [60]. Similar to DT, the PLX-mediated depletion is reversed within 7 days after discontinuing treatment and returning the rodent to standard chow diet.

Another pharmacological method of myeloid cell depletion is administration of clodronate liposomes [61]. The phagocytic microglia and macrophages engulf the liposomes and internalize chlodronate, a bisphosphonate which induces cell apoptosis and myeloid cell depletion without affecting the nonphagocytic cells. However, in addition to myeloid cells, other phagocytic cells of the reticuloendothelial system including dendritic cells can be depleted, thus confounding interpretation of the study results. Clodronate liposomes can be administered by intraperitoneal injection to achieve systemic macrophage depletion or intravitreally to achieve local depletion of phagocytic cells in the retina [46, 62, 63]. The liposomes have to be continuously administered (typically every 3–4 days) to achieve sustained depletion, which is not possible for intravitreal administration.

Pharmacological inhibition or genetic deletion of C–C motif chemokine ligand 2 (CCL2, also called monocyte chemoattractant protein 1, MCP1) or its receptor C–C motif chemokine receptor 2 (CCR2) ablate monocyte recruitment but spare non-classical monocytes that do not express CCR2 [63].

While informative, experiments that involve depletion strategies are limited by the fact that all microglia/macrophage cell subtypes are depleted creating a confound in which the effects of depletion on a particular subtype cannot be distinguished from the effects of depletion on other subtypes. This can lead to inaccurate conclusions about the role of microglia/macrophages in a particular disease or condition.

## Myeloid cells in acute ischemic retinopathies and neurodegeneration

Ischemic retinopathies including diabetic retinopathy, retinopathy of prematurity, ischemic optic neuropathy, and central retinal artery or retinal vein occlusions, are major causes of blindness (Table 1). Ischemia causes a lack of oxygen and nutrients to the retinal tissue, which leads to injury and cell death. Reperfusion, or the return of blood flow, can further exacerbate injury by promoting oxidative stress and inflammation. The underlying pathophysiology of ischemic retinopathies is complex and not fully elucidated. However, various mechanisms such as inflammation, oxidative stress, and apoptosis and other forms of cell death are reported to play a role in ischemic retinopathies [64, 65].

**Table 1:** Prevalence of human ischemic retinopathies

Disease	Frequency in the US	Study
Ischemic optic neuropathy	2.3 to 10.2 per 100,000	Raizada et al. (2024) [66]
Central retinal artery occlusion	1 per 100,000	Farris et al. (2024) [67]
Central retinal vein occlusion	8 per 10,000	Blair et al. (2023) [68]
Diabetic retinopathy	26.43% of diabetics	Lundeen et al. (2023) [69]
Retinopathy of prematurity	125,000 ROP per 23 million births	Bhatnagar et al. (2023) [70]

**Fig. 6:**
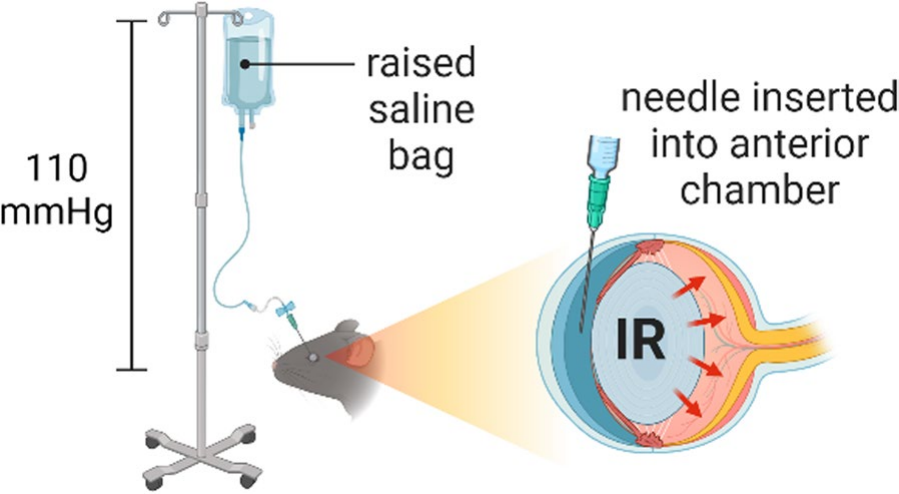
Illustration of the retinal ischemia–reperfusion (IR) injury model

The rodent model of retinal ischemia–reperfusion (IR) injury is widely utilized as an acute model in which to investigate the pathophysiology of ischemic retinopathies [71]. The IR model is achieved by cannulating the anterior chamber of the eye using a needle connected to an elevated saline bag to elevate the intraocular pressure (IOP) (Fig. 6). The IOP is raised above the systolic blood pressure to restrict the retinal blood flow and cause ischemia. The needle is then removed after a period of time (40 to 90 min with longer times leading to a more profound injury) to allow reperfusion, which subsequently triggers inflammation and neuronal degeneration [72].

We and others have shown that acute retinal ischemia leads to neurovascular injury and progressive cell death by apoptosis, necroptosis and other forms of cell death [46, 73, 74]. Concurrently, there is activation and proliferation of microglia, non-parenchymal macrophages (such as perivascular macrophages), and recruitment of blood-borne (infiltrating) monocytes [46, 73, 75]. These cells play either a protective or deleterious role after retinal injury depending on their molecular profile and activation state. Our publications and others recently introduced the concept that myeloid cells including microglia and macrophages can exhibit a pro-resolving phenotype and play a protective role in retinal injury. This protective effect is conferred by upregulation of reparative molecules in retinal ischemic injury and removal of optic nerve myelin debris in nerve crush/degeneration models [46, 51, 76, 77].

Myeloid cells play a critical role in the response of the retinal tissue to ischemia. Following ischemic injury and neurovascular damage, microglia undergo activation, resulting in changes in their morphology and gene expression, followed by proliferation and migration. Blood-borne monocytes are recruited to the injured retina and contribute to the retinal immune response to injury [78–80]. Microglia and the infiltrating macrophages rapidly become activated and migrate to the site of injury/ischemia. For example, IR injury induces a dramatic increase of microglia/macrophages CD68^+^ cells in the inner retina by day 1, which become amoeboid-appearing by 3 days after ischemia [81]. A rapid increase in Iba-1^+^ microglia /macrophages in the ganglion cell layer also is observed between 1 and 14 days post IR [73, 82]. The activation of myeloid cells may confer either protective or detrimental effects on the outcome of retinal IR as these cells release a wide range of cytokines, which can mitigate or exacerbate the underlying condition. The impact of the myeloid cells on retinal neurons after IR depends on the immunological milieu, which can be affected by the time course of injury as well as the presence or absence of infiltrating monocytes [46, 73, 79, 80]. Intriguingly, pre- and post-ischemic conditioning strategies that involve brief periods of ischemia before or after a damaging ischemic insult, have demonstrated neuroprotective effects in the retina [83–85]. In the context of cerebral ischemia, microglia have been identified as contributors to this protection [86]. However, the precise mechanisms by which microglia exert their protective effects in response to retinal ischemic conditioning are largely unknown.

In our studies, systemic macrophage depletion using clodronate liposomes led to increased neurodegeneration and retinal hemorrhage after IR suggesting both neuronal and vascular protective effects of infiltrating myeloid cells in this model [46]. Microglia depletion in other models showed conflicting results. One study showed no difference in ganglion cell degeneration after microglia depletion in a mouse model of optic nerve crush [62]. Other studies argue for neuroprotection after microglia depletion in mouse models of autoimmune uveitis and NMDA-induced excitotoxicity [87, 88]. Microglia ablation using Cx3cr1CreER × B6-iDTR (TG) mice or PLX-5622 appears to increase photoreceptor death in retinal detachment models, suggesting a protective role of microglia [89].

**Fig. 7:**
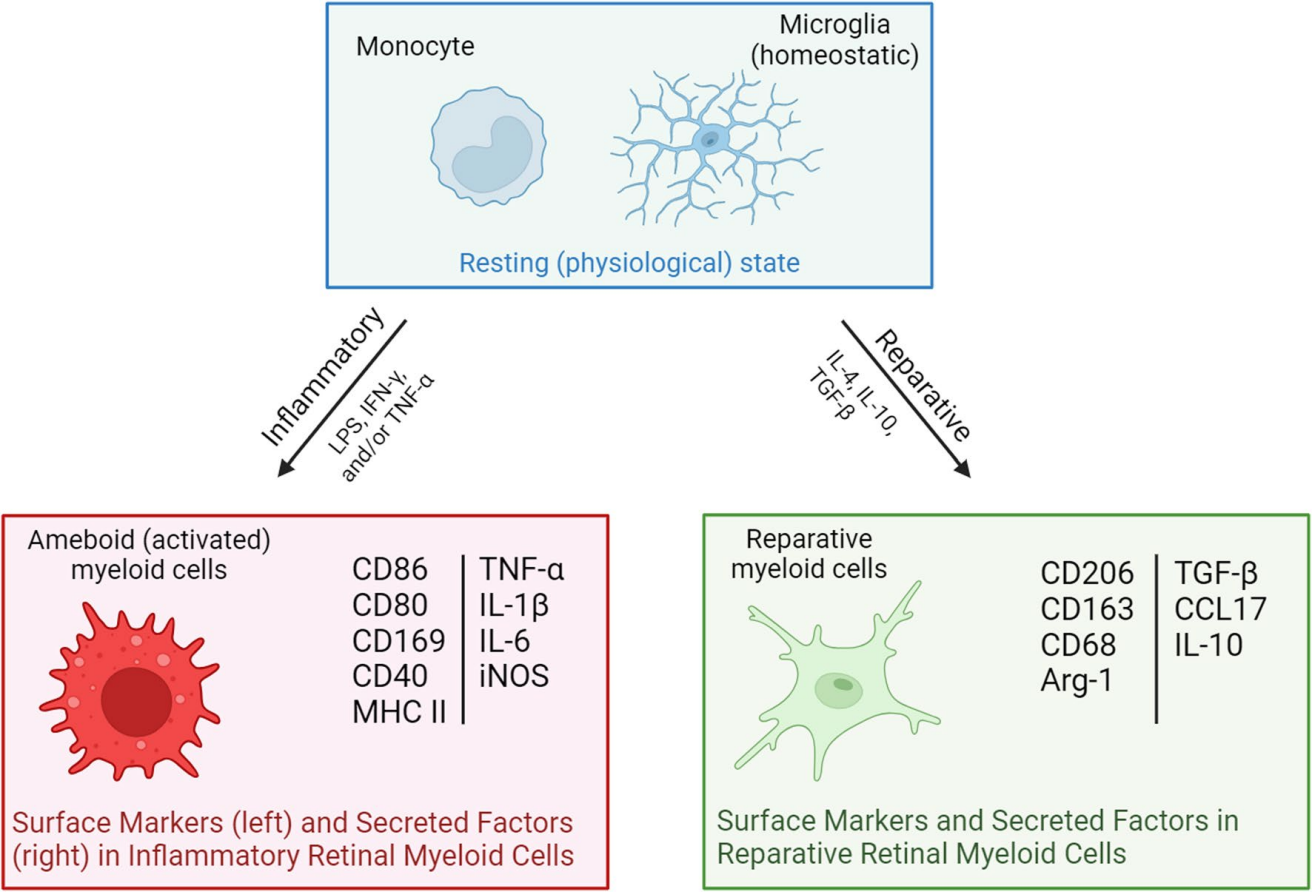
Phenotypical differences, surface markers, and secreted factors of inflammatory and reparative myeloid cells. CCL17: C–C motif chemokine ligand 17, IL-10: interleukin 10, IL-1β: interleukin 1 beta, IL-4: interleukin 4, IL-6: interleukin 6, iNOS: inducible nitric oxide synthase, LPS: lipopolysaccharide, MHC II: major histocompatibility complex class II, TNF-α: tumor necrosis factor alpha

Earlier investigations showed that microglia and macrophages can exhibit either pro-inflammatory (M1) or reparative (M2) phenotypes and undergo functional switching between two phenotypes depending on the local environment [90]. However, it is now accepted that the phenotypic status is not binary, but rather it is a spectrum and intrinsic or extrinsic factors can induce changes from one end of the phenotypic spectrum to the other (Fig. 7 and Table 2) [91]. M1 phagocytes are implicated in neuronal degeneration and dysfunction of the neural network due to their production of pro-inflammatory cytokines and mediators. Conversely, M2 phagocytes are thought to inhibit inflammation and promote tissue remodeling by altering gene expression and the secretion of neuroprotective factors [92, 93]. The M1 phenotype is typically induced extrinsically by the presence of pro-inflammatory cytokines, interferons (IFNs), and interleukins (IL). The M1 phenotype also can be induced intrinsically by oxidative stress. During the inflammatory process, M1 myeloid cells release proinflammatory cytokines that can influence the phenotypic profile of surrounding myeloid cells. The M2 classification can be subdivided into M2a, M2b, M2c and M2d subcategories. The M2a phagocytes are activated by IL-4 and IL-13, whereas M2b are primarily activated by immune complexes. Interleukin 10 (IL-10), transforming growth factor beta (TGF-β), and glucocorticoids induce the M2c phenotype whereas M2d is induced by interleukin 6 (IL-6) and adenosine [94, 95]. These activators of the M2 phenotype rely on signal transduction mediated by the transcription 6 (STAT6) pathway [96, 97]. STAT6 regulates the transcriptional activities of anti-inflammatory genes including mannose receptor 1 (CD206) and the enzyme arginase 1 (Arg-1) [98].

**Table 2:** Chart of inflammatory and reparative myeloid cell markers and their functions

Markers		Function	
Inflammatory		Reparative	
CD80	Antigen presentation	CD68	Phagocytic marker
CD86	Co-stimulation	CD163	Scavenger receptor
CD40	MHC II stimulation	CD206	Mannose receptor
iNOS	Nitric oxide production	Arg-1	Neuroprotection, T cell suppression
IL-1β	Pro-inflammatory cytokine	IL-10	Anti-inflammatory cytokine
IL-6	Pro-inflammatory cytokine	TGF-β	Anti-inflammatory cytokine
TNF-α	Pro-inflammatory cytokine	CCL17	Chemokine for T helper 2 (Th2) cells
MHC II	Antigen presentation		
Sialoadhesin (Sn, CD169)	Cell–cell interactions		

Myeloid cells respond to retinal ischemia by secreting cytokines. According to a recent study, microglia activation after retinal ischemia triggers a cytokine and Toll-like receptor response in the IR murine model by two hours after ischemia [99]. During the acute phase of injury, activated microglia release pro-inflammatory mediators, including tumor necrosis alpha (TNF-α) and interleukin 1 beta (IL-1β), through activation of the inflammasome which can contribute to tissue damage [100–102]. Studies have shown that IL-1β expression is upregulated and maintained up to 7 days during the acute phase of IR injury [103]. Similar to IL-1β, TNF-α is upregulated in retinal tissue during the acute phase leading to a form of necrotic death known as necroptosis [104, 105]. In contrast, myeloid cells also can release anti-inflammatory mediators such as IL-6, IL-10 and TGF-β, which contribute to the resolution of inflammation, dampening of oxidative injury, and enhancement of tissue repair after injury [106–108]. This anti-inflammatory myeloid cell phenotype can be achieved pharmacologically by drugs including the antibiotic, minocycline, or by genetic manipulation. Studies have shown that minocycline treatment shifts microglia polarization to an M2 -like phenotype with increased expression of interleukin 4 (IL-4) and earlier restoration of BRB function [73, 75]. In our hands, we have shown that a drug form of the enzyme Arg-1 suppresses the myeloid cell inflammatory response and promotes neurovascular protection. The protective effects of Arg-1 were mediated by myeloid cells since myeloid-specific deletion of Arg-1 exacerbated the retinal injury outcomes [46].

**Fig. 8:**
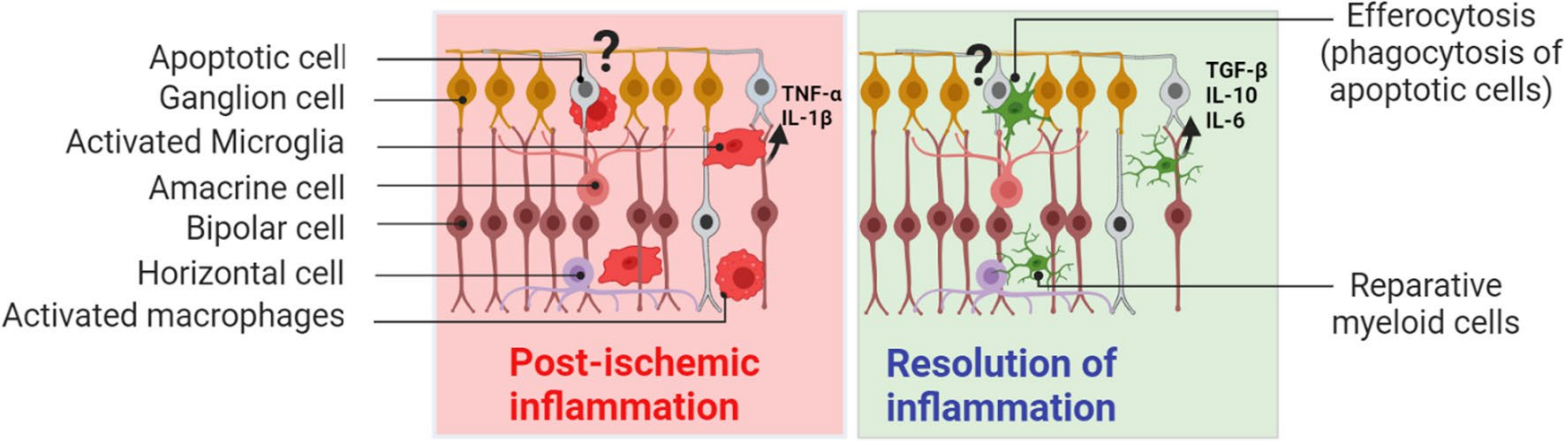
Myeloid cell response to retinal ischemic injury. Illustration of the myeloid cell inflammatory response (left) and reparative response (right) to IR. In the left panel, ameboid microglia secrete pro-inflammatory cytokines. In the right panel, secretion of anti-inflammatory cytokines and efferocytosis by myeloid cells can help to resolve inflammation. Although the myeloid cell phagocytic response is reported in the inner retina after ischemic injury, its roles in pathology progression and retinal outcomes are unknown, as denoted by the question mark

In addition to cytokine release, myeloid cells can phagocytose cellular debris and apoptotic cells by a programmed process termed efferocytosis [73]. Studies examining efferocytosis in the retina have focused primarily on photoreceptor degeneration with conflicting results documenting both reparative and deleterious roles of myeloid cell phagocytosis [89, 109–111]. In the inner retina, microglia/macrophage mediated phagocytosis has been described in retinal IR injury, optic nerve crush, and ocular injury models but its mechanistic role in injury progression is unclear [73, 75]. There is evidence that activated microglia/macrophages frequently associate with and engulf apoptotic neurons across multiple retinal layers after IR injury. This was shown by accumulation of the Iba-1^+^/CD68^+^ marker that designates microglia phagocyte phenotype, enveloping RGC following IR injury [75]. The authors suggested that microglia and monocyte-derived macrophages can phagocytose dying RGC and displace amacrine cells in the GCL, as well as synapses in the IPL. These findings imply that microglia/macrophage -mediated phagocytosis may play a key role in the response to IR injury in the retina via engulfing apoptotic cells (Fig. 8). A more mechanistic understanding of the pathways that mediate the metabolic reprogramming of efferocytic macrophages will provide insight into the regulation of efferocytosis and its impact on retinal outcomes after ischemic insult. This knowledge also could guide the development of novel treatment strategies designed to harness the benefits of efferocytosis to improve outcomes in diseases associated with retinal neuroinflammation and neurodegeneration.

## Myeloid cells in diabetic retinopathy

The disease of diabetic retinopathy (DR) occurs in 30% to 40% of individuals with diabetes mellitus and is the major cause of blindness in working adults. The early stage of DR, also called non-proliferative DR, presents with neuronal and microvascular dysfunction and endothelial cell damage secondary to chronic hyperglycemia. The later, sight-threatening stage is called proliferative DR since it is characterized by abnormal vessel growth (neovascularization) secondary to microvascular dysfunction and ischemia. The clinical and pathological features of DR have been the focus of excellent recent reviews [112–114].

The involvement of microglia/macrophages in the neurovascular loss in the diabetic retina is well established. However, the exact molecular mechanisms underlying the contribution of the microglia/macrophages to the neurovascular degeneration and repair in DR are unclear. It is recognized that myeloid cells are activated and increase in number in the diabetic retina of murine models of type 1 and type 2 diabetes [115, 116]. In response to DAMPs, microglia change from a highly ramified form to an amoeboid phenotype with retracted processes. The activated myeloid cells can modulate the pathological progression of DR by the production of cytokines or by direct interaction with other cell types in the retina [23, 24].

The microglia-mediated inflammatory response can be attenuated pharmacologically by drugs such as minocycline [117]. A recent report showed that microglia undergo necroptosis in DR secondary to hyperglycemia [118]. In this study, the authors used a pharmacological inhibitor of necroptosis and concluded that inhibiting necroptosis of microglia ameliorated retinal neuroinflammation and neurodegeneration, thereby suggesting a protective role of the microglia. However, the authors did not quantify the microglia number after inhibiting necroptosis and the reported protective effect could have resulted from direct inhibition of necroptosis in other retinal cell types. Another set of recent studies looked at microglial necroptosis in a mouse model of oxygen-induced retinopathy [119]. The authors identified a subpopulation of microglia that highly expressed necroptosis-related genes including *Rip3*. After activation by hypoxia, these microglia were committed to necroptosis through a RIP1/RIP3 -mediated pathway, which also induced release of fibroblast growth factor 2 (FGF2) from the microglia. Microglia-specific deletion of *Rip3* markedly attenuated the retinal neovascularization caused by hypoxia, thereby implicating this necroptotic pathway in the retinal neovascular response.

Chemokines are a family of proteins that are involved in recruitment and activation of leukocytes to the site of injury. Chemokines are upregulated in DR and result in increased monocyte and neutrophil recruitment to the diabetic retina. The recruited monocytes bind to adhesion glycoproteins including vascular adhesion protein-1 (VAP-1) and ICAM-1 on the endothelial cell surface and subsequently extravasate to the site of injury [120], increasing vascular permeability [113]. Interestingly, vascular endothelial growth factor (VEGF) a highly expressed angiogenic factor implicated in DR, upregulates ICAM-1 to promote the adherence and infiltration of monocytes [120]. Other chemokines contribute to the growing inflammatory state. CCL2 is upregulated after DR and has been implicated in its progression as it binds to its receptor, CCR2, on monocytes to increase monocyte infiltration and vascular leakage [121, 122]. However, other molecules participate in a protective compensatory response to limit injury to the diabetic retina. For example, CX3CR1 is a receptor for the chemokine fractalkine (CX3CL1 or FKN) that is constitutively expressed by microglia. CX3CR1-FKN signaling is regarded as protective since its deletion leads to a robust microglial activation and enhanced inflammatory response. Conversely, intravitreal treatment with recombinant soluble FKN improves vascular integrity and ameliorates perivascular microglial clustering and the progression of DR [123, 124]. Interestingly, transient depletion and repopulation of microglia in the diabetic murine retina is neurovascular protective due to microglial reprogramming and proliferation of a homeostatic microglial phenotype. These authors propose that the newly repopulated microglia proliferate from a homeostatic microglia pool that supports neuronal and vascular integrity [125].

Interestingly, studies using myeloid cell-specific knockout and transgenic (over-expressing) mice suggest that microglia can be steered to a protective phenotype in DR. A recent study focused on peroxisome proliferator-activated receptor alpha (PPARα), a transcription factor that regulates lipid metabolism, highlights this development. These authors reported that microglia-specific deletion of PPARα in streptozotocin (STZ)-induced DR resulted in microglial mitochondrial dysfunction, altered lipid metabolism, and increased activation leading to exacerbated retinal pericyte loss and neuronal dysfunction. Phenotypically, the retinal microglia from the knockout mice showed enlarged soma and retracted processes. In contrast, microglia-specific overexpression of PPARα in STZ-induced DR attenuated retinal dysfunction and reduced pericyte loss [126]. Similarly, other authors reported that myeloid-specific deletion of the histone demethylase, Kdm6a, ameliorates retinal inflammation, thinning, and visual impairment in type 1 diabetic Akita mice. This study showed that Kdm6a in microglia/macrophages aggravates diabetic retinopathy by increasing lipocalin 2 (LCN2), which impairs glycolysis in photoreceptor cells [127]. This finding concurs with our recent report of a decreased overall glycolytic response in the diabetic mouse retina revealed by Seahorse analysis [128]. These studies emphasize the important role of myeloid cells in the pathogenesis of DR and the potential of using reparative gene modulation to employ them as therapeutic envoys.

Myeloid-derived cells can participate in the pathogenesis of DR not only by promoting neoangiogenesis as discussed later, but also by compromising blood flow to the retina [35]. As the resident myeloid-derived cells of the retina, the microglia have an essential role in signaling between the retinal vasculature and neurons to maintain the neurovascular unit. The microglia regulate the retinal circulation by mediating capillary constriction in a CX3CR1-FKN dependent manner and capillary constriction only occurs at points of microglia-capillary contact [35]. The same authors noted a loss of retinal blood flow and reduced capillary diameter by 4 weeks in the rat STZ-induced model of DR, which coincided with increased microglial-capillary contact in the diabetic retina [35]. Notably, in addition to animal models of DR, retinal blood flow is reportedly reduced in diabetic patients [129, 130]. Imaging studies of the retinal circulation in patients using flouroscein angiography reported lower blood flow in patients with insulin-dependent type 1 diabetes compared to nondiabetic subjects prior to evidence of DR, suggesting abnormalities of the retinal circulation may be an early event in the pathogenesis of DR that could be mediated by myeloid cells.

## Myeloid cells in retinal pathological angiogenesis

Although the role of retinal glial cells in retinal neovascularization has been a focus of recent studies, relatively less is known about the contribution of myeloid cells to this abnormality. The late stage of DR, also called proliferative DR, involves pathological angiogenesis and the formation of abnormal and leaky blood vessels that interfere with vision. Considering the fact that murine models of diabetic retinopathy don’t develop proliferative DR, researchers have used other murine models of ischemic retinopathies including oxygen-induced retinopathy (OIR) as a proxy to understand the role of myeloid cells in diabetic retinal angiogenesis. The murine model of OIR initially described by Smith et al. is regarded as a historical and reproducible model in which to study the pathological angiogenesis that occurs in retinopathy of prematurity (ROP) and proliferative DR [131]. Using this model involves placing mouse pups and their dams in a hyperoxia chamber (75% oxygen) on postnatal day 7 (P7) for as long as 5 days. The animals are then transferred to room air (21% oxygen) on postnatal day 12 (P12). The exposure to different oxygen levels triggers the vaso-obliteration of the central retinal vessels between P7-P12, which is followed by vascular regrowth between p12 and p17 and pathological retinal neovascularization at P14-P17 [131, 132].

In the OIR model, retinal myeloid cells exhibit an M1-like “inflammatory” phenotype on P13 within a day after transfer back to room air and then shift to an M2-like “anti-inflammatory” phenotype between P14 and P21 [133]. Another study confirmed the increase of M2 macrophages around neovascular tufts at P17. This study implicated M2 macrophages in pathological neovascularization as selective depletion of M2 macrophages with intravitreal mannosylated clodronate liposomes promoted physiological revascularization. In contrast, the intravitreal injection of bone marrow-derived M2 macrophages promoted pathological retinal angiogenesis [134]. PLX-5622 -mediated microglia depletion reduced pathological angiogenesis in OIR [135]. Systemic macrophage depletion with intraperitoneal clodronate liposomes reduced retinal avascular area and neovascular tufts at P17 after OIR [136]. Similarly, the selective depletion of vitreal macrophages by intravitreal injection of clodronate liposomes reduced retinal neovascularization in OIR [137]. However, a recent study reported the opposite results with intravitreal injection of clodronate liposomes increasing vessel obliteration and subsequent neovascularization [138].

A recent study utilizing single-cell RNA-Seq (scRNA-Seq) to better understand the complexity and heterogeneity of the macrophages in pathological angiogenesis identified several microglia subtypes and showed the proximity of these cells to the pathological neurovascular tufts [139]. A transcriptome analysis of myeloid cells revealed 11 transcriptionally distinct clusters of cells collected from OIR retinas at P17. After comparing the different lists of gene markers, the authors identified these clusters as subtypes of microglia, macrophages, and monocytes. They further showed that a cluster of hyperproliferative microglia were localized next to pathological tufts in OIR retinas. This cluster showed a gene signature characteristic of chromatin modification with upregulation of class I histone deacetylases (HDAC 1, 2 and 3), suggesting that chromatin modification–mediated epigenetic regulatory mechanisms may underlie the upregulation of proliferative markers and cell cycle–related genes in these cells.

Importantly, there is evidence that therapeutic modulation of myeloid cells can achieve reparative angiogenesis. For example, inhibition of myeloid cell infiltration and activation by the glucagon-like peptide-1 receptor (GLP-1R) agonist, NLY01, decreased pathologic retinal neovascularization [140]. Similarly, we reported that Arg-1, an enzyme that catalyzes the breakdown of arginine, limits neurovascular injury in the OIR mouse model and this effect may relate to its modulation of myeloid cell phenotype. In this study, administration of pegylated arginase 1 (PEG-Arg-1) enhanced vascular repair and attenuated pathological neovascularization whereas Arg-1 deletion worsened pathological angiogenesis and increased retinal injury [141]. Interestingly, a study also revealed a massive 60-fold upregulation of arginase-1 mRNA in retinal microglia/macrophages in the OIR model, which was the highest increase in of all gene transcripts included in the RNAseq analysis [142]. Interestingly, PEG-Arg-1 treatment increased macrophage/microglial cells that strongly expressed the M2-like macrophage-microglia marker CD206 and the M1-like marker CD16/32. This double-positive M1/M2-like phenotype is characteristic of the angiogenic phenotype reported earlier in the OIR model. Interestingly, myeloid-derived VEGF and hypoxia-inducible factor (HIF1α) have not been implicated in OIR neovascularization [143]. A follow up study by the same group showed that myeloid cell-specific depletion of Von Hippel–Lindau tumor suppressor protein (pVHL) which targets HIF factors for rapid proteosomal degradation in normoxic conditions leads to stabilization of myeloid-derived HIFs and ameliorates OIR-induced neovascularization [144]. In contrast to the neuroprotective effect of minocycline reported earlier in DR, minocycline treatment reduced microglial reactivity and physiological vascularization as evident by increased avascular area, suggesting that a treatment could have opposite effects in different retinal injury models [145].

Crosstalk between retinal neuronal cells and macrophages also may contribute to the neoangiogenic response in DR. One study has implicated the neuropilin-1 (NRP-1) receptor in neuronal-macrophage crosstalk, which is an obligate receptor that binds ligands involved in signaling pathways implicated in cell attraction, migration and survival [146]. Using the mouse model of OIR, the authors determined that NRP-1 positive macrophages were selectively recruited to the site of pathological retinal neoangiogenesis in mouse retinas in response to the local production by neurons of the NRP-1 ligand, semaphorin 3A, in addition to tissue production of VEGF. The level of semaphorin 3A was elevated in vitreous samples collected from diabetic patients with late-stage DR, further implicating it as a crucial chemoattractant that may recruit proangiogenic macrophages to the site of retinal neurovascular injury in the innate immune response to OIR [146].

Myeloid derived pro-angiogenic cells (PAC), formerly known as endothelial progenitor cells, also were implicated recently in vascular homeostasis. PACs represent a subset of monocyte- derived cells with monocytic and hematopoietic surface markers [27]. An investigation of the functional differences between PACs isolated from the blood of healthy controls and type 2 diabetic patients with and without DR showed a shift in the production of angiogenic factors by myeloid-derived cells in diabetic patients. The authors noted a strong increase of the proangiogenic factor S100A8 that is constitutively expressed in immune cells but a decrease of VEGF-A [147]. The underlying effects of the altered PAC differentiation in relation to angiogenesis in diabetes need further exploration.

## Microglia/macrophages in human ischemic retinopathy

Research on the specific roles of microglia and macrophages in human ischemic retinopathy is limited to descriptive and correlative studies. However, observations from in vivo related models suggest they play a significant role in the pathogenesis of this disease. For example, macrophages and microglia are purportedly recruited to the retinal surface and inner retinal layers in human conditions of retinal ischemia such as diabetic retinopathy [148, 149]. In these studies, macrophage-like cells (MLCs) including microglia, perivascular macrophages, monocyte-derived macrophages, and/or vitreal hyalocytes were observed at the retinal surface (vitreoretinal interface). These cells are abundant in proliferative DR and can be imaged in human subjects or rodent models with retinal vascular inflammation using optical coherence tomography [148, 149]. Additionally, immunolabeling studies performed by our laboratory and others on human donor retinal sections post-mortem show an increase in microglia number in DR retinas [141, 150]. These microglia exhibit a hypertrophic phenotype compared to the ramified morphology of microglia in nondiabetic retinas. The further observations that microglia cluster around the retinal vasculature, and assemble around retinal ganglion cell bodies and their axons, suggest a direct myeloid cell response to DR-induced neurovascular injury [141, 150]. These correlative findings suggest that microglia and macrophages may contribute to ischemic retinopathies observed in the clinic, but more definitive research is needed to determine the extent to which findings from animal models of DR will translate to humans.

In this regard, current clinical interventions targeting ischemic retinopathies including DR involve anti-VEGF or steroid injections, laser photocoagulation therapy, vitrectomy and other invasive treatments that do not restore vision but only slow disease progression [151]. Despite the promising results in experimental models of retinal ischemia that minocycline and other FDA-approved drugs can induce a reparative phenotype in myeloid cells, these studies have yet to be translated to the clinic. Interestingly, although experimental studies have shown that targeting microglia with GLP-1R agonists has the potential to decrease retinal degeneration and neovascularization in ischemic retinopathies [140, 152], clinical trials show conflicting results with recent studies correlating GLP-1R agonists administration with worsening of DR [153–155]. However, it remains unclear whether this finding reflects a direct effect of GLP-1R agonists on the retinal microglia or may be caused by the acute reduction in blood glucose and rapid weight loss attributed to these drugs. Regardless, this study highlights the complexities of translating therapeutics that target myeloid cells in retinal ischemia from experimental animals to clinical practice.

## Conclusions and unanswered questions

This review has highlighted the multifaceted and dynamic involvement of myeloid cells in retinal neurovascular degeneration and angiogenesis. Although the actions of these cells can be either detrimental or reparative, identifying the key regulatory pathways by which myeloid cells contribute to retinal injury and healing and devising strategies to steer them toward a pro-regenerative phenotype remains crucial for therapeutic development. We have included key studies reviewed in this article in a table for ease of access (Table 3). Building upon this initial knowledge is warranted to bridge the gap between preclinical research and clinical practice. Repurposing existing FDA-approved medications in clinical trials for other indications represents a compelling strategy to accelerate the translation of novel treatments for ischemic retinal injuries from bench to bedside. By harnessing the multifaceted potential of myeloid cells and optimizing treatment approaches, we can advance therapeutic strategies for neurovascular pathologies with significant clinical impact.

**Table 3:** Summary of Key Studies Highlighting the Role of Myeloid Cells in Ischemic Retinopathies

Study	Model	Species	Outcomes and measures
Acute ischemic retinopathy			
Wagner et al. (2021) [99]	IR	Rat	Microglial activation enhances pro-inflammatory cytokines accompanied by TLR signaling 2 h post-IR (p = 0.028)
Abcouwer et al. (2021) [75]	IR	Mouse	Myeloid leukocyte accumulation 14 days post-IR (p ≤ 0.001)
Tang et al. (2020) [82]	IR	Mouse	Elevated Iba1^+^ cells numbers in retina 1-day post-IR (p < 0.001)
Fouda et al. (2018) [46]	IR	Mouse	Myeloid arginase 1 deletion exacerbates loss of retinal neurons (p < 0.05) and thinning of the retina at 7 days post-IR (p < 0.05)
Ahmed et al. (2017) [73]	IR	Mouse	Increased number of IBA1^+^ cells/mm in GCL at 1-day post-IR (p ≤ 0.0001)
Sanchez et al. (2003) [108]	IR	Rat	Increased expression of IL-6 by microglia/macrophages at 2 h post-IR (p < 0.05)
Diabetic retinopathy			
Huang et al. (2023) [118]	SIDR	Mouse	Retinal microglia necroptosis promotes neuroinflammation in the early stages of diabetic retinopathy
Park et al. (2021) [116]	SIDR	Rat	Increased Iba1^+^ retinal microglia density in diabetic mice compared to control mice (p < 0.05)
Atawia et al. (2020) [115]	WDIDR	Mouse	Arginase 2 (A2) deletion inhibits retinal microglia activation (p < 0.05)
Mendiola et al. (2016) [124]	Ins2^Akita^	Mouse	Recombinant fractalkine reduces perivascular microglial clustering (p < 0.01)
Cardona et al. (2015) [123]	Ins2^Akita^	Mouse	Retinal microglia increase in diabetic mice (p < 0.05)
Rangasamy et al. (2014) [121]	SIDR	Rat	CCL2 activates retinal microglia and attenuates vascular leakage and infiltration of monocytes
Krady et al. (2005) [117]	SIDR	Rat	Microglia activate in the early stages of diabetic retinopathy
Pathological retinal angiogenesis			
Liu et al. (2022) [156]	OIR	Mouse	Localization of microglia near pathological neovascular tufts (p < 0.001)
Liu et al. (2022) [138]	OIR	Mouse	Macrophage depletion by clodronate increases neovascularization on P12 (p < 0.05)
Zhou et al. (2022) [135]	OIR	Mouse	Microglial depletion suppresses pathological neovascularization (p < 0.05)
He et al. (2021) [119]	OIR	Mouse	Microglia necroptosis largely ablates retinal angiogenesis
Liu et al. (2020) [142]	OIR	Mouse	Arginase-1 transcript is elevated in microglia/macrophages (p < 0.001)
Villacampa et al. (2020) [144]	OIR	Mouse	Depletion of myeloid-cell specific Vhl stabilizes HIFs and stimulates retinal revascularization (p < 0.05; p < 0.001)
Zhu et al. (2017) [133]	OIR	Mouse	M2 macrophage polarization genes are upregulated on P13-P24 (p < 0.05)
Gao et al. (2016) [136]	OIR	Mouse	Macrophage depletion by clodronate reduces percentage of neovascular area (p < 0.01)
Zhou et al. (2015) [134]	OIR	Mouse	Bone marrow-derived M2 macrophages increase pathological neovascularization (p < 0.01)
Dejda et al. (2014) [146]	OIR	Mouse	NRP-1^+^ myeloid cells are recruited to neovascularization sites in the retina
Zhou et al. (2015) [134]	OIR	Mouse	Bone marrow-derived M2 macrophages increase pathological neovascularization (p < 0.01)
Kataoka et al. (2011) [137]	OIR	Mouse	Depletion of native vitreal macrophages reduces neovascularization (p < 0.01)
Human ischemic retinopathy			
Ong et al. (2021) [149]	DR	Human	Increased density of macrophage-like cells in PDR compared to healthy patients (p < 0.05)
Zeng et al. (2008) [150]	DR	Human	Microglial cells were observed to cluster around veins in the GCL, microaneurysms, and retinal and vitreal neovascularization

CCL2: Monocyte chemoattractant protein-1, DR: Diabetic retinopathy, FABP5: Fatty acid-binding protein 5, GCL: Ganglion cell layer, HIF: Hypoxia-inducible factor, IR: ischemia reperfusion, NRP-1: Neuropilin-1, OIR: Oxygen-induced retinopathy, PDR: proliferative diabetic retinopathy, PKM2: enzyme pyruvate kinase M2, SIDR: Streptozotocin-induced diabetic retinopathy, TLR: Toll-like receptor, Vhl: Von Hippel-Lindau tumor suppressor protein, WDIDR: Western-diet induced retinopathy

Despite the wealth of studies on the role of myeloid cells in retinal ischemia, the following questions are some of the mysteries that remain unanswered and need to be addressed in future research:

1. Do microglia and macrophages differ in their response to injury in terms of cytokine release and phagocytic activity?
2. Is phagocytosis reparative or deleterious to outcomes of inner retinal injury and can the myeloid cell phenotype be therapeutically modulated to promote injury resolution?
3. Does the myeloid cell phagocytic response depend on the dying cells and thereby differ between various retinal degenerative conditions?

## Data Availability

Not applicable.

## Abbreviations

Arg-1: Arginase 1
BRB: Blood retinal barrier
BBB: Blood–brain barrier
CAM: Capillary-associated microglia
CCL17: C–C motif chemokine ligand 17
CNS: Central nervous system
CX3CR1: Chemokine fractalkine receptor 1
CCL2: Chemokine ligand 2
CSF1R: Colony-stimulating factor-1 receptor
DAMPs: Damage-associated molecular patterns
DR: Diabetic retinopathy
DT: Diphtheria toxin
DTR: Diphtheria toxin receptor
ELM: External limiting membrane
ECM: Extracellular matrix
Fpr2: Formyl peptide receptor 2
GCL: Ganglion cell layer
GLP-1R: Glucagon-like peptide-1 receptor
HIF1α: Hypoxia-inducible factor
ILM: Inner limiting membrane
INL: Inner nuclear layer
IPL: Inner plexiform layer
ICAM-1: Intercellular adhesion molecule-1
IFNs: Interferons
IL: Interleukin
IL-1β: Interleukin 1 beta
IL-6: Interleukin 6
IL-10: Interleukin 10
IOP: Intraocular pressure
IR: Ischemia–reperfusion
JVM: Juxtavascular microglia
LCN2: Lipocalin 2
LPS: Lipopolysaccharide
MLCs: Macrophage-like cells
MHC II: Major histocompatibility complex class II
MCP-1: Monocyte chemoattractant protein 1
MPs: Mononuclear phagocytic cells
NFL: Nerve fiber layer
NRP-1: Neuropilin-1
NVU: Neurovascular unit
ONL: Outer nuclear layer
OPL: Outer plexiform layer
OS: Outer segment
OIR: Oxygen-induced retinopathy
PAMPS: Pathogen-associated molecular patterns
PVM: Perivascular macrophages
PPARα: Peroxisome proliferator-activated receptor alpha
PECAM-1: Platelet-endothelial cell adhesion molecule-1
PAC: Pro-angiogenic cells
RGCs: Retinal ganglion cells
RPE: Retinal pigment epithelium layer
ROP: Retinopathy of prematurity
TAM: Tamoxifen
Th2: T helper 2 cells
STAT6: Signal transducer and activator of transcription 6
TGF-β: Transforming growth factor beta
TNF-α: Tumor necrosis alpha
VAP-1: Vascular adhesion protein-1
VCAM-1: Vascular cell adhesion molecule-1
VEGF: Vascular endothelial growth factor
